# How fern and fern allies respond to heterogeneous habitat — a case in Yuanjiang dry-hot valley

**DOI:** 10.1080/19420889.2021.2007591

**Published:** 2021-12-13

**Authors:** Feng-Chun Yang, Chaya Sarathchandra, Jing-Xin Liu, Hua-Ping Huang, Jian-Yong Gou, Ye Li, Xiao-Ye Mao, Hui-Ting Wen, Jun Zhao, Ming-Fu Yang, Suthathong Homya, Kritana Prueksakorn

**Affiliations:** a Yibin Vocational and Technical College, Yibin, Sichuan, China; b School of Chinese Medicinal Material Resources, Guangdong Pharmaceutical University, Guangzhou, China; c Yi Minority Culture Research Center of the Key Research Base of Philosophy and Social Sciences of Sichuan Province; d State Key Laboratory of Urban and Regional Ecology, Research Center for Eco-Environmental Sciences, Chinese Academy of Sciences, Beijing, China; e Department of Biological Science, Faculty of Applied Sciences, Rajarata University of Sri Lanka, Mihintale, Sri Lanka; f Environmental Education Center, Xishuangbanna Tropical Botanical Garden, Chinese Academy of Sciences, Mengla, Yunnan, China; g Environment and Plant Protection Research Institute, Chinese Academy of Tropical Agricultural Sciences, Haikou, China; h Honghe Meteorological Bureau, Yunnan, China; i Yanyuan Vocational Middle School of Liangshan Yi Autonomous Prefecture, Sichuan, China; j Department of Environmental Science, Faculty of Science and Technology, Phuket Rajabhat University, Phuket, Thailand; k Faculty of Environment and Resource Studies, Mahidol University, Nakhon Phathom, Thailand

**Keywords:** Diversity, dry-hot valley, fern, indicator, rarity

## Abstract

The Yuanjiang dry-hot valley features hot and dry climate, low vegetation and soil degradation. It had lush vegetation in the past, but has become degraded in recent decades. Understanding the interrelationship between species and the habitat is necessary to explain this change. In this study, a link between fern and fern allies – a group that is hypersensitive to environmental factors and their circumstances is constructed. Intensive transects and plots were designed to be proxies for extant fern and fern allies, and their habitats. Fifty years of meteorological records of precipitation and temperature along altitude and river running direction (latitudinal) were employed. Alpha and beta diversity are used to access diversity. Species_estimated, Singletons, Uniques, ACE, ICE, and Chao2, which associate to abundance and rarity, are subscribed to the correlation between fern and fern allies, and their ecosystem. Eight species, *Selaginella pseudopaleifera, Aleuritopteris squamosa, Adiantum malesianum, Pteris vittata, Davallia trichomanoides, Sinephropteris delavayi, Selaginella jugorum*, and *Lygodium japonicum* are used as indicators of a typical xeric and sun-drying habitat. The results indicate (1) accompanied by dramatically shrinking habitats, fern and fern allies are in very low diversity and abundance, whereas the rarity is relatively high; (2) for fern and fern allies, environmental factors are positive when altitude goes up; and (3) eight indicator species are latitudinally correlated with fern and fern allies along the river running direction.

## 1. Introduction

The dry-hot valley is represented by high temperature and dry air throughout the year, and it is one of the arid ecosystems in the world influenced by climate change [1–3]. Climate change has resulted in extensive alteration of terrestrial ecosystems, including the change in biogeographic distribution and biological diversity, and the interaction between organisms and their living circumstances [4]. Understanding the interrelationship between plants and their environmental conditions in arid ecosystems is a preliminary step to interpret the mechanism of adaptation, or giving a feasible solution for saving earthbound environment [5].

Yuanjiang dry-hot valley is located in the upper and middle region of Yuanjiang-Red River, an international river shared by China and Vietnam [6]. In the valley, the nature of soil, water and fertilizer are not in good condition [7,8]. The vegetation has changed gradually in the past 500 years, but has been degraded significantly in recent decades causing a dry and hot tropical ecosystem [9]. Broadleaf and evergreen plants have been shrinking, while it is currently dominated by montane savannah, covered with few trees and high grasses [10]. To elucidate this change, it is necessary to understand a corresponding pattern between plants and environmental factors such as humidity, temperature and water availability. Fern and fern allies are traditionally considered hypersusceptible to environmental changes; their occurrence or disappearance is closely correlated to their living place [11]. In this study, the relationship between fern and fern allies and environments in the Yuanjiang dry-hot valley is investigated together with species population and environment factors.

## 2. Materials & methods

### 2.1. Transect and plot designing

**Figure 1.**
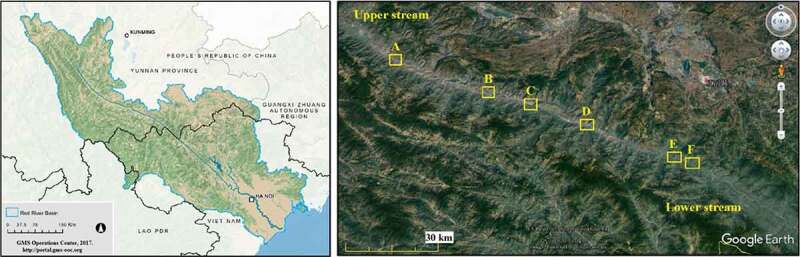
Watershed of Yuanjiang-Red River and transects A-F in the dry-hot valley

**Figure 2.**
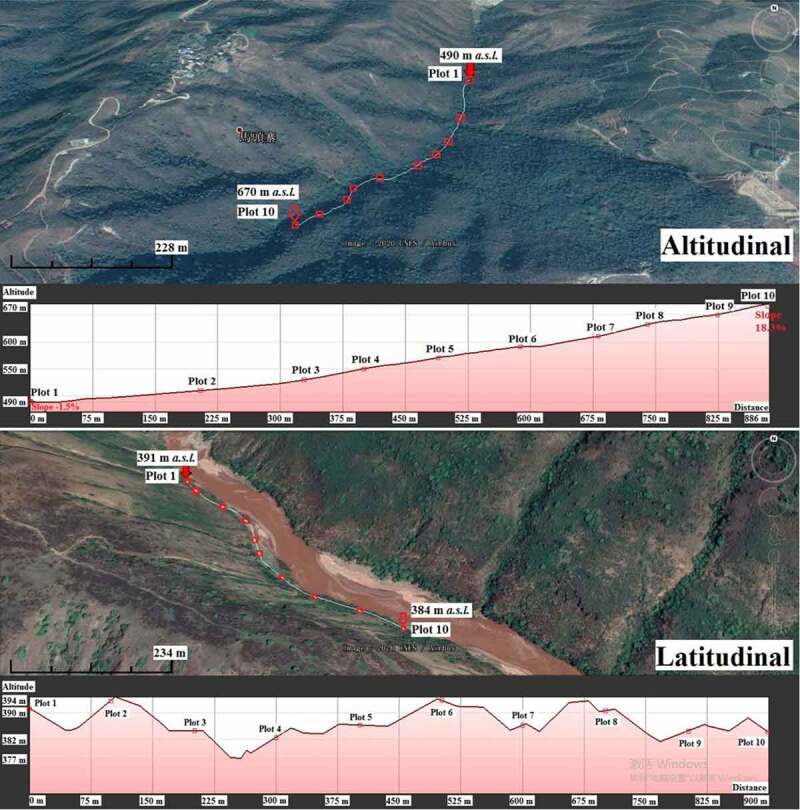
Transects designing along altitude and latitude

The research site is the core area of Yuanjiang dry-hot valley with a length of 105.26 km along the river channel, oriented northwest to southeast (Figure 1a). Six transects were arranged randomly (Figure 1b) with a concept of no interference by human activities, where three transects were set up altitudinally along a mountain slope, and the other three latitudinally along the riverside (Table 1). Altitudinal transects were 180 m in height vertically, with ten plots arranged equidistantly 20 m apart from the lower plot to the higher. The latitudinal transects were 900 m in length along the river channel, and ten plots were spaced at a distance of 100 m evenly (Figure 2). Plots were 2 m × 2 m squared. All fern species in the sampling sites were investigated in summer from May to August, 2017 and 2018, periods when average monthly temperature and precipitation are mostly stable [12]. The nomenclature follows *Flora Yunnanica* [13].

**Table 1.** Location, altitude and circumstance, and orient of each transect

Tr.	Location	Habitat	Orient
A	23°28′05.22″N, 102°10′43.32″E	360 ± 15 m *a.s.l*., open river terrace	Latitudinal
B	23°19′13.98″N, 102°32′45.12″E	490 ~ 690 m *a.s.l*., closed ravine	Altitudinal
C	23°17′17.03″N, 102°39′59.50″E	440 ~ 640 m *a.s.l*., open ravine	Altitudinal
D	23°11′51.97″N, 102°53′57.82″E	260 ± 15 m *a.s.l*., closed river terrace	Latitudinal
E	23°04′29.90″N, 103°11′40.64″E	360 ~ 560 m *a.s.l*., closed ridge	Altitudinal
F	23°03′20.57″N, 103°16′38.56″E	230 ± 15 m *a.s.l*., closed river terrace	Latitudinal

Tr.: Transect

### 2.2. Meteorological data collecting

Meteorological records, primarily annual temperature and precipitation, were collected from long-time meteorology stations in the Yuanjiang dry-hot valley at 312 m to 1758 m *a.s.l*. (Table 2). Temperature and precipitation are considered the main environmental factors correlated to fern and fern allies growth and distribution in the dry-hot valley. Linear regression is conducted based on twenty stations.

**Table 2.** The annual average temperature and precipitation at different altitudes in Yuanjiang dry-hot valley (1962–2018)

Code	Station	Altitude (m *a.s.l.)*	Temperature (^O^C)	Precipitation (mm)
1	Yuanyang (Nansha)	312	23.9	788
2	Yuanjiang	401	24.5	738
3	Yuanjiang farm	542	23.9	984
4	Nansa	576	23.5	747
5	Hongguang farm	675	22.8	894
6	Dong e	775	22.3	888
7	Ganzhuang	821	22.8	923
8	Honghe	974	20.8	785
9	Majie	1051	19.1	1219
10	Niujiaozhai	1209	19.1	1677
11	Fengchunling	1263	18	2159
12	Panzhihua	1337	18.4	1944
13	Leyu	1510	17.6	1093
14	Damang	1530	18.1	1235
15	Yuanyang (Xinjie)	1635	16.3	1670
16	Baohe	1696	16.4	970
17	Jiayan	1712	16.7	1246
18	Nanuoyunhai	1745	16.7	1308
19	Nanuo	1752	16.8	1312
20	Dayangjie	1758	17.3	1211

### 2.3. Statistical analyses

Alpha and beta biodiversity (*α*- and *β*-index) are both employed. The *α*-index, associating to species, is commonly represented by Shannon-Wiener index (*H*), Evenness (*J*), and Dominance. The *β*-index, which is based on not only species occurrence but also abundance analytically, and associated to living habitat, is represented by *Bray-Curtis* [14, 15; 16]. When comparing *turnover* and *nestedness* components [17], it is more approachable to use *Bray–Curtis* similarity index for analysis of abundance and incidence, following the strategy of Diserud and Ødegaard [18].

Additionally, there are approaches to assess the difference in plots and transects using the program EstimateS Version 9.1 [19]. *Species_estimated, Singletons, Uniques, ACE, ICE*, and *Chao2* are prevalently accepted in diverse studies, such as savanna ants of Australia [20], the elevational richness of Colorado Mountain [21] and many different areas [22–24], with details as follows: 1) *Species_estimated* indicates the expected number of species in *t* pooled plots. It is not a real but a calculated index. Increase of species approaches zero early or late indicating different habitats; 2) *Singletons* indicates the number of species with only one individual in *t* pooled plots. It is associated with the species rarity; 3) *Uniques* indicates the number of species that occurs in only one plot in *t* pooled plots regardless of the population size; 4) *ACE* is an abundance coverage-based estimator of species richness. It is performed on the basis of species with 10 or fewer individuals in the sample. It is a diversity metric that involves an arbitrary threshold for abundance; 5) *ICE* is an incidence coverage-based estimator of species richness. It is performed based on species found in 10 or fewer sampling units; 6) *Chao2* is a species richness estimator based on the incidence. This means that it requires data on the presence and absence of a species in a given sample [24–26]. These six indices are applied in this study, and their model inferences are tested on Chi-square statistic (*χ^2^*) at 0.05 significance level. Residuals are evaluated for normality using the Shapiro-Wilk test (normality was assumed when *P ≥ 0.5*) [27]. With Tukey’s method, multiple comparisons are used to test the disparity between habitats at 0.05 significant levels.

Therefore, to cope with the issues mentioned above, two kinds of species matrices are required. The first matrix is individual-based, while the second matrix is occurrence-based ignoring the number of individuals. All matrices are computed 999 times in EstimateS (version 9.10) and then the selected index is rearranged and analyzed with packages *vegan* [28], *labdsv* [29] and *lme4* [30] in R (v.2.15.2) [31]. Indicator value function (*IndVal*) in package *indicspecies* [32] is also employed. *IndVal* is a random variable that takes “1” to represent a happened event and “0” for nothing. After that, multilevel pattern analysis is conducted at significance level *P*= 0.05 in the pooled species.

## 3. Results

The analytical results are from 2016 fern individuals belonging to 17 genera and 13 families (Table 3).

**Table 3.** Species in transects. (following Flora Yunnanica)

Family	Species	Tr. A	Tr. B	Tr. C	Tr. D	Tr. E	Tr. F
Selaginellaceae	*Selaginella biformis*	0	10	0	0	0	20
Selaginellaceae	*Selaginella delicatula*	0	8	0	0	0	60
Selaginellaceae	*Selaginella jugorum*	0	20	0	0	0	0
Selaginellaceae	*Selaginella pseudopaleifera*	163	2	0	0	0	0
Selaginellaceae	*Selaginella stauntoniana*	20	0	0	0	0	0
Selaginellaceae	*Selaginella doederleinii*	0	0	0	0	0	2
Selaginellaceae	*Selaginella uncinata*	0	0	0	0	1	0
Selaginellaceae	*Selaginella helferi*	0	8	0	0	0	40
Equisetaceae	*Equisetum hyemale*	0	0	0	20	0	20
Lygodiaceae	*Lygodium japonicum*	0	7	0	0	0	0
Dennstaedtiaceae	*Hypolepis punctata*	0	0	0	1	0	0
Dennstaedtiaceae	*Microlepia speluncae*	0	1	0	0	0	0
Pteridaceae	*Pteris vittata*	0	35	10	30	4	140
Pteridaceae	*Pteris excelsa*	0	1	0	0	0	0
Pteridaceae	*Pteris grevilleana*	0	10	0	0	0	0
Pteridaceae	*Pteris fauriei*	0	1	0	0	0	0
Pteridaceae	*Pteris linearis*	0	0	0	1	0	0
Pteridaceae	*Pteris tripartita*	0	20	0	0	0	0
Pteridaceae	*Pteris ensiformis*	0	3	0	0	0	0
Pteridaceae	*Histiopteris incisa*	0	3	0	0	0	1
Sinopteridaceae	*Aleuritopteris squamosa*	220	100	20	0	0	0
Sinopteridaceae	*Doryopteris ludens*	0	50	0	0	0	0
Adiantaceae	*Adiantum malesianum*	0	320	0	30	1	18
Adiantaceae	*Adiantum philippense*	0	1	0	0	0	0
Adiantaceae	*Adiantum capillus-veneris*	0	0	0	1	0	0
Hemionitidaceae	*Pityrogramme calomelanos*	0	0	0	1	0	0
Thelypteridaceae	*Cyclosorus parasiticus*	0	0	0	1	0	0
Aspleniaceae	*Sinephropteris delavayi*	0	300	0	0	0	0
Nephrolepidaceae	*Nephrolepis auriculata*	0	20	0	0	0	0
Davalliaceae	*Davallia trichomanoides*	0	0	0	0	0	150
Polypodiaceae	*Pyrrosia tonkinensis*	0	0	0	50	0	20
Polypodiaceae	*Phymatosorus scolopendria*	0	11	0	0	0	0
Polypodiaceae	*Phymatosorus cuspidatus*	0	40	0	0	0	0

Tr. = Transect

### 3.1. Spatial patterns of thermal and moisture in Yuanjiang dry-hot valley

**Figure 3.**
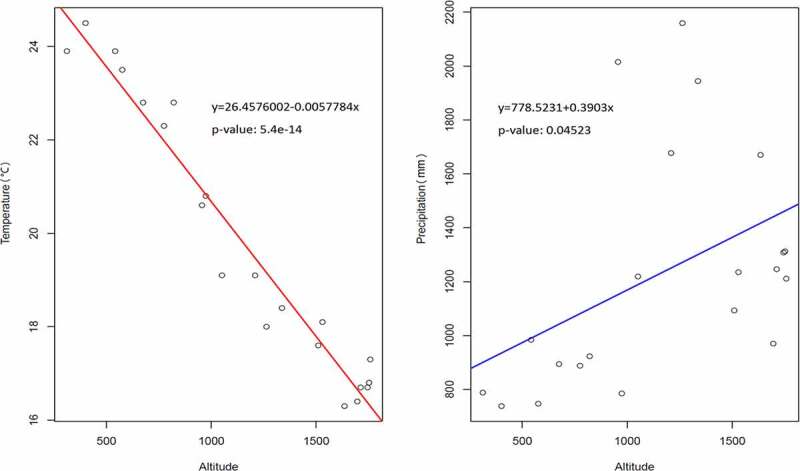
Correlation of temperature and precipitation in altitudal gradients

Temperature and precipitation are the two main climatic factors being considered in this study. According to a 57 year (1962–2018) data profile, the temperature decreases when altitude increases (Figure 3). The maximum annual temperature was 24.5°C at 401 m *a.s.l*. while the minimum was 16.3°C at 1,635 m *a.s.l*. (Table 2). Conversely, precipitation rose when altitude increased (Figure 3). The maximum annual precipitation was 2,159 mm at 1,263 m *a.s.l*. while the minimum was 738 mm at 401 m *a.s.l*. (Table 2).

Rainfall in Yuanjiang dry-hot valley depends on two factors. The first is the process of wet air sinking, pressurizing and heating-up when warm and humid airflow moves down to the valley, which weakens the uplifting of water vapor in the precipitation system and reduces water vapor condensation [10]. Secondly, typical valley wind is generated by consistent sunshine, high temperature and rapid evaporation. Water vapor is taken to a relatively high altitude by the up-valley wind and then freezes and falls to the ground in the forms of droplets [33]. Therefore, the valley’s weather condition is cool and humid at the relatively high altitude, but it is reversed in the lower place. Fifty year records in the Jinsha River valley display a similar statistical profile [10]. Dry air and high temperature prevail as typical environmental features in the dry-hot valley.

### 3.2. Shannon-Wiener index (H) and diversity variation along mountain slope and riverine separately

**Figure 4.**
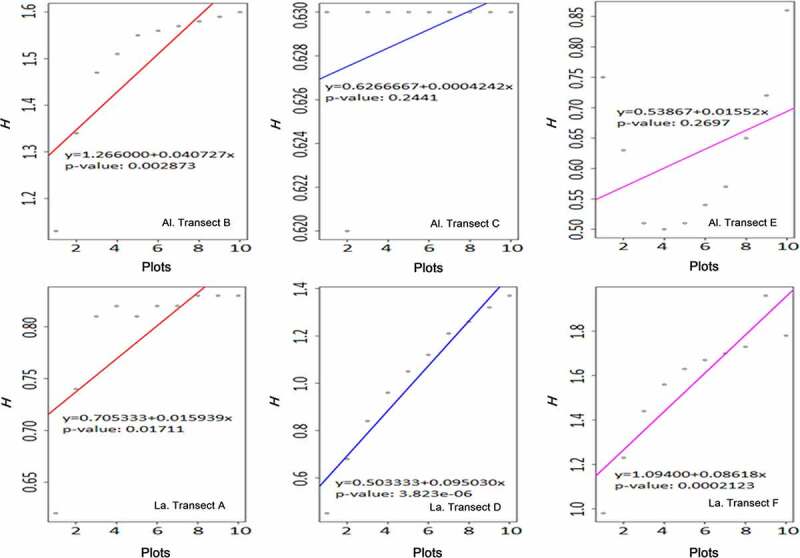
The variance of Shannon-Wiener index (*h*) in transects. (Al. = Altitudinal, La. = Latitudinal)

Shannon-Wiener index among plots in each transect are displayed separately in Figure 4 with a general linear regression. The changes along plots can be noticed, and the habitat heterogeneity can also be identified based on these dispersing values.

#### 3.2.1. *Altitudinal transects (mountain slope*, Figure 4, *B/C/E)*

Plots in transect are labeled 1–10 from the bottom to the top of the mountain. Transect B was a closed ravine while C was open. Transect E was a mountain ridge. In general, there was a positive correlation between the *H* index and the altitude. It clearly shows a tendency that fern and fern allies became more diverse as the altitude increased.

Transect B had a significant correlation (*P*< 0.05). It was a ravine with a closed habitat and vegetative plants. The humidity inside the ravine was relatively high due to well irrigating from the upper site. Most fern and fern allies grew well in this circumstance.

Transect C was also a ravine but aggregated by fragment rocks, and poor vegetation. It could not keep groundwater, neither a close and humid habitat. Fern and fern allies were rarely discovered in this site.

Transect E was a mountain ridge which could not maintain water consistently, nor construct a humid habitat. Xerophytes bushes were well developed on the ridge compared to fern and fern allies.

#### 3.2.2. *Latitudinal transects (Riverside*, *Figure 4*, *A/D/F)*

Plots in transects were labeled 1–10 from the upper to the lower across 900 meters. Transect A was an open river terrace while D and F were closed. The *H* index was positively and significantly correlated along the river (*P< 0.05*). Compared to altitudinal transects, the water supply of transects A/D/F seemed sufficient because it was close to the river channel. Simultaneously, closed habitats were constructed in some areas in transect D and F due to developed shrubs and trees. However, transect A did not develop flourishing riverine vegetation. The main reason would be ascribed to irregular river tides.

### 3.3. β-diversity and the habitat heterogeneity in The Valley

#### 3.3.1. In transects

Although all the six transects existed in a narrow valley of 105 km in length, the *Bray-Curtis* index showed a deviation altitudinally and latitudinally (Table 4). The maximum was 0.292 in latitudinal transect D and F which defined similar living circumstances of fern and fern allies. The minimum was 0 in A and D, A and F, and A and E which defined different circumstances.

**Table 4.** Similarity in transects

	A	B	C	D	E	F
**A**		0.148	0.092	0	0	0
**B**			0.059	0.108	0.01	0.11
**C**				0.125	0.222	0.039
**D**					0.073	**0.292**
**E**						0.02
**F**						

A, D, and F, latitudinal transects; B, C, and E, altitudinal transects.

#### 3.3.2. In plots

To specify the microhabitat in the plots of each transect, *Bray-Curtis* indices were calculated. The values are listed separately (Table 5). Transects A, B, D, and F were similar in the plots with *Bray-Curtis*, and the living circumstance of fern and fern allies was continuous. In contrast, Bray-Curtis in the plots of transects C and E was approaching 0. This is due to the poor records of target species and the same low humidity in these two places. The results match well with the field investigation.

**Table 5.** Similarity in plots

Transect A											Transect B										
	**1**	**2**	**3**	**4**	**5**	**6**	**7**	**8**	**9**	**10**		**1**	**2**	**3**	**4**	**5**	**6**	**7**	**8**	**9**	**10**
**1**		0.8	0.59	0.55	0.53	0.57	0.59	0.65	0.44	0.76	**1**		0.82	0.84	0.89	0.85	0.81	0.54	0.22	0.37	0.29
**2**			0.44	0.41	0.39	0.42	0.44	0.49	0.32	0.86	**2**			0.88	0.87	0.77	0.68	0.46	0.19	0.46	0.39
**3**				0.82	0.65	0.68	0.67	0.75	0.56	0.54	**3**				0.88	0.8	0.71	0.47	0.2	0.47	0.36
**4**					0.82	0.87	0.82	0.81	0.72	0.51	**4**					0.85	0.73	0.51	0.21	0.4	0.31
**5**						0.95	0.92	0.83	0.88	0.48	**5**						0.8	0.5	0.21	0.36	0.29
**6**							0.93	0.85	0.83	0.52	**6**							0.68	0.39	0.43	0.34
**7**								0.78	0.8	0.52	**7**								0.67	0.5	0.39
**8**									0.73	0.6	**8**									0.36	0.41
**9**										0.4	**9**										0.72
**10**											**10**										
**Transect C**											**Transect D**										
	**1**	**2**	**3**	**4**	**5**	**6**	**7**	**8**	**9**	**10**		**1**	**2**	**3**	**4**	**5**	**6**	**7**	**8**	**9**	**10**
**1**		0	0	0	0	0	0	0	0	0	**1**		0.09	0.08	0.08	0.02	0.06	0.06	0.06	0.06	0.06
**2**			0.73	0	0	0	0	0	0	0	**2**			0.8	0.67	0.06	0.27	0.29	0.24	0.27	0.27
**3**				0	0	0	0	0	0	0	**3**				0.86	0.09	0.38	0.4	0.33	0.38	0.38
**4**					0	0	0	0	0	0	**4**					0.12	0.47	0.5	0.42	0.47	0.47
**5**						0	0	0	0	0	**5**						0.27	0.25	0.26	0.24	0.22
**6**							0	0	0	0	**6**							0.8	0.93	0.77	0.69
**7**								0	0	0	**7**								0.82	0.88	0.8
**8**									0	0	**8**									0.86	0.79
**9**										0	**9**										0.92
**10**											**10**										
**Transect E**											**Transect F**										
	**1**	**2**	**3**	**4**	**5**	**6**	**7**	**8**	**9**	**10**		**1**	**2**	**3**	**4**	**5**	**6**	**7**	**8**	**9**	**10**
**1**		0	0	0	0	0	0	0	0	0	**1**		0.7	0.79	0.4	0.64	0.58	0.59	0.47	0.26	0.22
**2**			0	0	0	0	0	0	0	0	**2**			0.64	0.43	0.54	0.5	0.5	0.43	0.2	0.17
**3**				0	0	0	0	0	0	0	**3**				0.4	0.6	0.51	0.47	0.34	0.26	0.22
**4**					0	0	0	0	0	0	**4**					0.34	0.32	0.38	0.35	0.16	0.13
**5**						0	0	0	0	0	**5**						0.76	0.63	0.56	0.26	0.23
**6**							0	0	0	0	**6**							0.56	0.49	0.23	0.19
**7**								0	0	0	**7**								0.83	0.46	0.44
**8**									0	0	**8**									0.51	0.5
**9**										0	**9**										0.57
**10**											**10**										

### 3.4. The variation of species_estimated, Singletons, Uniques, ACE, ICE, and Chao2

**Figure 5.**
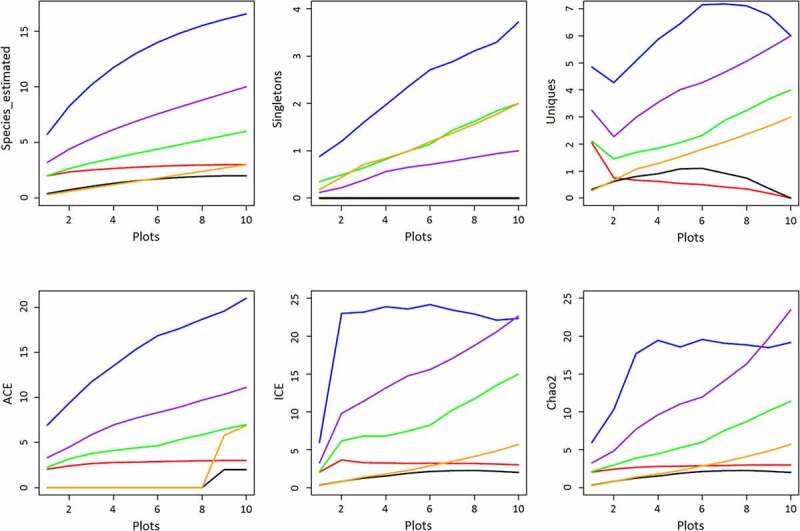
The variance of estimated species, rarity, and abundance in plots 1–10 of each transect. (Red = transect A; blue = transect B; black = transect C; green = transect D; Orange = transect E; purple = transect F)

***Species_estimated*** (Figure 5): Transects A and C approached a gentle level earlier than others. The estimated species curve reaches zero prematurely indicating a homogeneous habitat among the plots investigated. There were three species in transect A: *Aleuritopteris squamosa, Selaginella stauntoniana*, and *S. pseudopaleifera*; while there were two species in transect C, *Pteris Vittata* and *A. squamosa*. Species increase in transect A and C stopped at plots 3 and 2 separately. There were no new species discovered in the following plots. In contrast, species accumulation curves in the other four transects keep going up from plot 1 to plot 10, showing a high possibility to find new species in the next plot. This indicates a heterogeneity of habitat among the plots.

***Singletons*** (Figure 5): It was zero in transects A and C because no species grew as a single individual. In transect F, the Singletons approached 1, and there was only one species (*Histiopteris incise)* found in plot 4. Unlike transects A, C, and F, Singletons increased quickly without a turnover in transect B, D, and E. The maximum of Singletons happens in transect B bearing approximately nine species, *Pteris vittata, P. ensiformis, P. excelsa, P. fauriei, Lygodium japonicum, Selaginella delicatula, Adiantum philippense, Microlepia speluncae*, and *Phymatosorus scolopendria*. These species are generally distributed worldwide in a broad niche. However, none of them colonize the whole plot. Instead, other species take advantage. Extreme water reliance takes charge of this. Water suspended in the air and absorbed in the living substance is vital to the growing and regeneration process.

***Uniques*** (Figure 5): According to the curves, transects A and C were in low *Unique*. Transect A was riverine; plots in this transect had similar habitats. Transect C was a ravine along a mountain slope, and it featured fragmented rocks and bare surface soil from plot 1 at the bottom to plot 10 at the top. Plots in this transect had similar habitats too. Therefore, there were very few species found in these two transects, and these species were presented in the plots from 1–10 evenly. In opposite to transects A and C, *Unique* increased in the plots of transects B, D, E, and F, indicating that more species and habitats were expected in the next plot. Transects D and F, for example, were riverine transects, the same with transect A, but the growing substance and canopy coverage made the habitat more suitable for fern and fern allies. When the investigated plots increased continuously, new records were found occupying a single plot or sharing a plot with other species, *e.g., Selaginella helferi, S. biformis, S. doederleinii, Histiopteris incisa*, and *Equisetum hyemale*.

***ACE, ICE*, and *Chao2*** (Figure 5): They verified each other by displaying similar curve shapes. Species richness in transect B, D, and F were much higher than other transects. The maximum richness was 22 species detected in transect B whereas only two species were detected in transect C.

### 3.5. Indicator species

**Figure 6.**
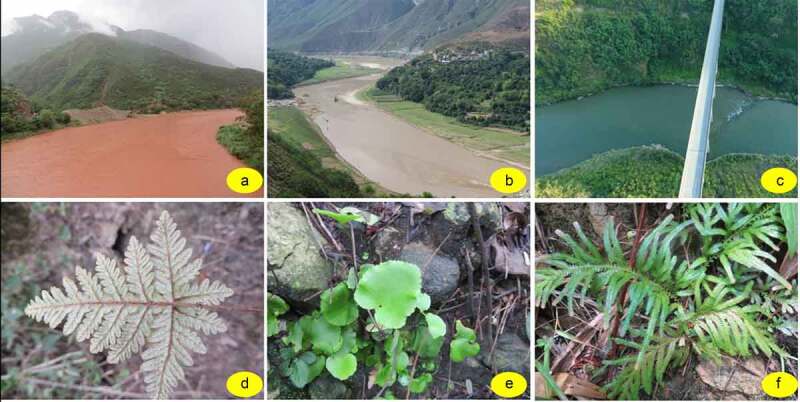
Indicator species in different segments of Yuanjiang dry-hot valley. (a) Upper stream, transect A; (b) Middle stream, transect B; (c) Downstream, transect F; (d) *Aleuritopteris squamosa* indicator of transect A; (e) *Sinephropteris delavayi* indicator of transect B; (f) *Selaginella pseudopaleifera* indicator of transect F

Eight species are screened out to represent the Yuanjiang dry-hot valley as shown in Table 6. All indicators perched at the altitudes 490 ~ 690 m although some of them distributed down to 260 m. All indicators require a humid environment although they grow in different river segments (Figure 6).

**Table 6.** Indicator candidates in Yuanjiang dry-hot valley

Code	Species	Altitude and habitat	Transect	*p*-value
1	*Selaginella pseudopaleifera*	360 ~ 690 m *a.s.l*., hot and wet	1,2	0.001 ***
2	*Aleuritopteris squamosa*	360 ~ 690 m *a.s.l*., hot and dry	1,2,3	0.001 ***
3	*Adiantum malesianum*	260 ~ 690 m *a.s.l*., hot, limestone	2,4,5,6	0.001 ***
4	*Pteris vittata*	260 ~ 690 m *a.s.l*., acidic soil	2,3,4,5,6	0.001 ***
5	*Davallia trichomanoides*	330 ~ 690 m *a.s.l*., hot and wet	6	0.001 ***
6	*Sinephropteris delavayi*	490 ~ 690 m *a.s.l*., hot and wet	2	0.002 **
7	*Selaginella jugorum*	490 ~ 690 m *a.s.l*., hot and wet	2	0.003 **
8	*Lygodium japonicum*	490 ~ 690 m *a.s.l*., dry acidic soil	2	0.018 *

## 4. Discussion

### 4.1. Variation of thermal and moisture altitudinally

Several studies have paid attention to altitudinal influence on vascular plants. In the tropical area, studies reveal a hump-shaped pattern with the highest diversity at mid-altitudes and then decrease toward both high and low altitudes [34]. The altitude where maximum fern and fern allies diversity occurs differs somehow among mountain ranges. For example, maximum diversity occurs around 1,800 m in both Costa Rica [35] and Mount Kinabalu, Borneo [36], 2,000 m in Bolivia [37], and 2,400 m on Mount Kilimanjaro, Tanzania [38]. Climatically, these gradients corresponded to the upper parts of tropical gradients where richness also declined. Species were comparable between these data sets at the same mean annual temperature. In temperate regions, richness was reported to decline continuously with elevation or remains roughly constant, such as New Zealand or North America [39]. In subtropical regions, a study from the Fanjingshan of Guizhou, China revealed a strong negative correlation between altitude and species [40].

The altitudinal transects along mountain slope were located from 360 ~ 690 m *a.s.l*. (Table 1). The annual temperature was over 22.3 ^O^C, and annual precipitation was less than 984 mm (Table 2). Species regeneration was difficult for most species. Generally, fern and fern allies were rarely discovered, in which two species were discovered in transect C, and three species were in transect E. Transect B was different from C and E. In contrast, it was rich in fern and fern allies with 22 species and 971 individuals (Table 3). Such a difference could be explained with topographic features. The altitude of transects C and E was around 970 m *a.s.l*., where annual temperature and precipitation were 20.8 ^O^C and 785 mm, respectively. In contrast, the altitude of transect B was around 1750 m *a.s.l*., where annual temperature and precipitation were 16.8 ~ 17.3 ^O^C and 1211 ~ 1312 mm, respectively (Table 2). Rainfall drained into the ravine and supports the plants inside. Water supply in transect B was better than others because there was plenty of water supply coming from the higher elevation. Relative humidity was recorded at above 90% in transect B, which was more humid than other transects (around 72%). Comparing to the drought in transects C and E, much more shrubs (*Salix myrtillacea, Ilex cornuta, Buddleja officinalis, Solanum verbascifolium etc*.) and trees (*Phyllanthus emblica, Trema tomentosa, Grewia biloba var. parviflora, Broussonetia papyrifera etc*.) were developed. A closed canopy was composed in dense vegetates. Diversity in the closed ravine was higher than an open ravine or a ridge.

### 4.2. Water condition determines the distribution of fern and fern allies

The population of species acquiring high water supply had shrunk in Yuanjiang dry-hot valley for centuries (Xu, 1985[9]). Undoubtedly, fern populations were distributed broadly in the past compared to the mosaic habitats nowadays. Water condition has traditionally been considered a decisive factor for fern and fern allies [35,41]. The fern and fern allies flourishment is interpreted as a reflection of environmental humidity [41] (or an optimal combination of mild temperature and humidity [35]. Compared to ground-living fern and fern allies, epiphytic species strongly rely on water vapor in the closed-canopy [42]. In this study, ground-living fern and fern allies did not display a different water requirement. Instead, both of them performed similar positive correlations to the water condition. It turns out that 1980 individuals (98%) were growing well in humid transects, i.e., A, B, D, and F (A, D, F benifited from river flow, while transect B benifited from inner cycling precipitation), whereas 36 individuals (2%) lived in dry transects. Considering transect B, the plot at the bottom preserved 54 individuals of 3 species. It increased to 364 individuals of 19 species at the top plot, almost seven times of the bottom. Plots in low altitude were occupied mainly by worldwide species such as *Pteris vittata* and *Adiantum malesianum*. However, it was altered to uncommonly seen species when the altitude went up, where the dominant species were *Sinephropteris delavayi* and *Phymatosorus cuspidatus*.

### 4.3. Heterogeneous habitat and fern and fern allies distribution

Generally, to maintain plants growing and population size, fern and fern allies have to absorb more water from the circumstance surroundings, e.g., *Pteris vittata, Lygodium japonicum*, and *Microlepia speluncae*. However, other species, such as *SelaginellaS delicatula, S. uncinata, Adiantum capillus-veneris*, and *A. malesianum*, survive in a unique approach, *e.g*., slender plant size and vegetative reproduction by rhizomes [41]. Their rarity in the plots is ascribed to frequent hot and dry air and habitat deterioration such as substance rocks fragmentation and surface soil erosion [9,43]. It has become worse in recent decades and has resulted in a dramatically declined environment. Reproductive organisms, e.g., spores, gemmae, and slender rhizomes, suffer and regeneration cannot proceed under such environmental stress. Habitat determines which plants grow, while environmental factors define a habitat. According to the habitat heterogeneity hypothesis [44,45], the living requirement differs in species, and each species has their living requirements exclusively. Their requirements include living substances, water, temperature, and nutrition, to name a few. A complicated and diverse habitat supports more species while a unitary habitat supports less. Diversity increases in more heterogeneous habitats [46]. For an arid ecosystem, disturbances with varying intensities and spatial scales are responsible for habitat patterns [47]. As regards the fern and fern allies distribution in Yunnan, China, heterogeneity was reported to be critical to the diversity of species and to be related to biogeographic zonation [48]. In Yuanjiang dry-hot valley, *Bray-Curtis* index revealed that transects C and E were unitary habitats, while transects A, B, D, and F were more complicated and diverse. In the field survey, only three species were recorded in the unitary transects C and E, whereas 33 species were found in heterogeneous transects A, B, D, and F (Table 3). A Topographic variation on altitudinal and latitudinal gradients and divergent temperature and water condition generated a mosaic microclimate on the slopes in the valley.

### 4.4. Fern and fern allies indicators

Indicator species are correlated to specific environmental factors. The population sizes usually increase or decrease due to the change of one or several factors. Their presence or absence is mostly determined by an environmental factor. They are optimal to indicate the environmental evolution process regarding their diverse habitat requirements in species [49–51]. Most fern and fern allies are strictly limited in their habitats because of their intrinsic sensitivity to environmental change. For example, *Gonocormus minutus* live on water due to their fragile mesophyll tissue, while *Selaginella tamariscina* can resist the extreme arid condition at Yang tribute due to its resuscitation, etc. They are considered key indicators of the environment [52].

In this study, screened indicators fit well with the hot-dry or hot-wet environment (Table 6). Three species were worldwide distributed with broad ecological amplitude, i.e., *Pteris vittata, Lygodium japonicum*, and *Adiantum malesianum*.

However, five other species were limited in distribution and confined to narrow amplitude, i.e., *Selaginella pseudopaleifera, S. jugorum, Sinephropteris delavayi, Davallia trichomanoides*, and *Aleuritopteris squamosa*. Although growing in different river segments from upper to lower, they all occurred in relatively humid areas and disappeared in hot and dry places. Similar results revealed the indicator function in these selected genera, e.g., endemic species of genus *Selaginella* in Philippine are recognized as indicators of refuge in the geological past [53] and *Davallia mariesii* is used as an indicator of landforms and vegetation types [54]. *A. squamosa* is endemic in the upper and middle segment of Yuanjiang-Red River, which strongly associates to dry-hot climate [55]. Meanwhile, a cryptic species *A. argentea*, is reported as a pyrophytic species and *Sinephropteris* is monotypic confined to karst in southwest China, northeast India, and north Burma [56].

## 5. Conclusions

In recent years, climate change has been broadly focused, while environmental disasters have occurred frequently. Significant land degradation and climate events have been reported for more than decades. Plants are seriously affected by environmental changes too. In response to such ecological crises, this research demonstrates such changes in Yuanjiang valley located in the first half of a river shared by China and Vietnam by focusing on fern and fern allies affected by other environmental factors. From bottom to top of the mountain, precipitation increased while the temperature decreased. Fern and fern allies are positively correlated with height as a result of atmospheric conditions. Species richness increases further accompanied habitat heterogeneity in the valley. Even with no influence of human disturbance, these current appearances are not the original pattern of the areas, indicating another consequence of climate change.
